# Maternal Aerobic Exercise during Pregnancy Can Increase Spatial Learning by Affecting Leptin Expression on Offspring's Early and Late Period in Life Depending on Gender

**DOI:** 10.1100/2012/429803

**Published:** 2012-09-16

**Authors:** Ayfer Dayi, Sinem Agilkaya, Seda Ozbal, Ferihan Cetin, Ilkay Aksu, Celal Gencoglu, Sultan Cingoz, Cetın Pekcetin, Kazim Tugyan, Berkant Muammer Kayatekin, Nazan Uysal

**Affiliations:** ^1^Department of Physiology, School of Medicine, Dokuz Eylul University, 35340 Izmir, Turkey; ^2^Department of Medical Biology and Genetics, School of Medicine, Dokuz Eylul University, 35340 Izmir, Turkey; ^3^Department of Histology and Embriology, School of Medicine, Dokuz Eylul University, 35340 Izmir, Turkey

## Abstract

Maternal exercise during pregnancy has been suggested to exert beneficial effects on brain functions of the offspring. Leptin is an adipocytokine which is secreted from adipose tissues and has positive effects on learning, memory, and synaptic plasticity. In this study, pregnant rats were moderately exercised and we observed the effects of this aerobic exercise on their prepubertal and adult offsprings' spatial learning, hippocampal neurogenesis, and expression of leptin. All the pups whose mothers exercised during pregnancy learned the platform earlier and spent longer time in the target quadrant. Their thigmotaxis times were shorter than those measured in the control group. It is shown that hippocampal CA1, CA3 neuron numbers increased in both prepubertal and adult pups, in addition that GD neuron numbers increased in adult pups. Leptin receptor expression significantly increased in the prepubertal male, adult male, and adult female pups. In our study, maternal running during pregnancy resulted in significant increase in the expression of leptin receptor but not in prepubertal female pups, enhanced hippocampal cell survival, and improved learning memory capability in prepubertal and adult rat pups, as compared to the control group. In conclusion, maternal exercise during pregnancy may regulate spatial plasticity in the hippocampus of the offspring by increasing the expression of leptin.

## 1. Introduction

It is known that aerobic exercise is necessary for human health. Regular aerobic exercise decreases body fat rate, increases skeletal muscle strength, improves respiratory capacity, and increases the HDL cholesterol in blood. In addition to the positive effects of the exercise on the body, it also has improving effects on brain functions [1]. It is shown that it increases cerebral angiogenesis and cerebral blood flow [2], capillary growth [3], and dendritic connections [4] and improves cognitive performance in young rats [5, 6]. Brain development in mammals begins intrauterine period and continues till the end of the adolescent period [7]. The brain may easily be affected by internal and external factors within this development process. It is observed that some events and factors encountered during this period have positive or negative effects on the brain development. For example, stressful situations such as maternal deprivation in the early development period may cause neuropsychiatric disorders such as anxiety disorders, schizophrenia, and depression [8, 9]. Besides, enriched environment and physical exercise increase the number of brain cells and improve learning and memory [10].

Hippocampus, which is one of the regions of the brains related to cognitive functions, is the center of spatial learning and memory [11]. Differently from the other regions of the brain, cells are more affected by external factors during their lifetime and situations such as exercise or enriched environment increase the number of neurons in this area [10, 12]. Psychological traumas such as stress also mostly influence the hippocampus in the brain [8, 12]. While the increase in the number of hippocampal cells enforces learning and memory, its decrease affects them negatively [13, 14]. 

Regular aerobic exercise during pregnancy is beneficial both for the mother and for the infant. It is observed that as a result of the exercise during pregnancy the muscle power and strength increase, excessive weight gain is prevented, as well as back pain, anxiety, and depression [15]. Other studies demonstrated that the aerobic exercise during pregnancy improves brain functions of the offsprings [16]. Hippocampal neurogenesis may continue lifelong under appropriate conditions, and especially enriched environment and physical exercise are seen to increase neurogenesis especially in gyrus dentatus (GD) in adults [13]. It is also shown in one of our previous studies that the exercise during the adolescent period increased hippocampal neurogenesis and spatial learning [14]. 

Leptin is a protein, the product of obesity (ob) gene. It is primarily produced in adipose tissue, and it provides nutrition and control of energy balance [17]. It is also thought to be produced and secreted locally in the central nervous system [18]. It is shown that leptin is carried by cerebrospinal fluid [19], enters into the brain by overcoming the blood brain barrier by means of the receptor, and is transferred to most regions of the brain by special carriers [20]. There is leptin receptor expression in many regions of the brain. In humans and rodents, leptin receptor mRNA is expressed in hypothalamus [21], cerebellum, hippocampus, and amygdala [22, 23]. Consequently it is thought that leptin may have an important effect on the brain development. It is indicated that leptin-deficient rats have smaller brain [24] and less myelin [25] and administering leptin to these rats corrects these abnormalities [26]. Leptin is also necessary for the regulation of locomotor activity and for neuronal and glial maturation in brain [27]. 

There is a limited number of studies concerning the effects of exercise on leptin, spatial learning, and memory. It is thought that the exercise regulates the glucose metabolism in brain, affects neurogenesis and neuroprotection, and regulates insulin, insulin-like growth factor-1 (IGF-1), and leptin signals in hypothalamus in order to control body weight [28, 29]. Leptin receptor- deficient rats (db/db) are obese and have insulin resistance [30]. Deficiency of dendritic branching and lack of hippocampal brain-derived neurotrophic factor (BDNF) are detected in these rats, and regression is observed in these findings when caloric limitation and voluntary exercise are implemented [31]. Besides, the relation between leptin, exercise, and hippocampus-related learning-memory has not been clarified yet.

Various studies revealed that maternal exercise during pregnancy increases hippocampus-related learning and memory in offsprings and improves spatial plasticity; however, it is still not known whether leptin has a role in this process or not. The aim of this study is to investigate the effects of maternal moderate aerobic exercise during pregnancy on hippocampus-related spatial learning, memory, the hippocampal neurogenesis, and hippocampal leptin receptor expression of offsprings in prepubertal and adult periods.

## 2. Material and Method

Fifty-six Wistar Albino rats were included in the study. The study group consisted of animals whose mothers performed regular aerobic exercise during pregnancy, while the control group consisted of pups whose mothers were sedentary throughout the pregnancy. Each group was further divided into subgroups: male, female, prepubertal (21 days old), and adult (120 days old), resulting in eight groups with 7 animals in each. Pregnancy was controlled by estrous followup. The animals were maintained under standard colony conditions with a 12 h light : 12 h dark cycle (lights on 07:00 h) at constant room temperature (23 ± 2°C) and humidity (60%), and food and water *ad libitum* throughout the experiments. Our study was carried out between 09:00 and 12:00 in a sound-attenuated, air regulated experimental room. All experiments were performed in accordance with the guidelines of the Experimental Animal Laboratory and approved by the Animal Care and Use Committee of the Dokuz Eylul University, School of Medicine. 

### 2.1. Regular Maternal Aerobic Exercise in Pregnancy

Mothers in the exercise group were familiarized to the treadmill, 1 week before the pregnancy, by 10 min/session a day at a speed of 5 m/min for 5 days. These mothers started exercising at 8 m/min for 30 min 5 days a week the day after mating. In the last week of pregnancy, the speed was reduced to 6 m/min and the exercise was done for 30 min 5 days a week. This exercise type is regular mild treadmill exercise [6, 32]. Learning tests were made when offsprings get 21 and 120 days old after birth. At the end of the 5-day learning tests, subjects were sacrificed and their brains were ejected for histological evaluation. 

### 2.2. Learning and Memory Evaluation

Learning and memory were evaluated by using Morris Water Maze (MWM). In tests, a black plexiglass pool having a diameter of 140 cm and a height of 75 cm was filled with warm water (22 ± 1°C). The platform having a 11 cm diameter, used within the pool, was 1.5 cm below the water surface. In all groups, the place of the platform was taught to them by making 5 trials for 4 days. The subject was expected to find the platform within 60 seconds after being placed in the water. It was allowed to stay on the platform for 20 seconds. The start point of the test was changed every day. Their duration of finding the platform, swimming speeds, and the distances covered within the 4-day learning process were calculated and evaluated. In the probe trial made on the 5th day, the platform was removed and the periods spent on the quadrant previously covering the platform and on the counter quadrant were evaluated for 60 seconds. The fact that the rat swam all around the pool wall at a distance of 15 cm in probe trial was evaluated as thigmotaxis which is an index of anxiety. 

HVS image video tracking system was used to make the records and analysis of learning tests.

### 2.3. Histological Evaluation

After the MWM experiments, each group of rats was sacrificed under ether anesthesia by removal of all of the blood in the heart. Brains were removed and fixed in 10% formalin in phosphate buffer for 24 h. The brains were sectioned coronally into sequential 6 *μ*m slices using a rat brain slicer. The number of neurons in each sample was estimated by taking three coronal sections through the hippocampus that corresponded approximately to plates 21, 23, and 25 in the rat atlas of Paxinos and Watson [33]. All sections were stained with cresyl violet. The images were analyzed using a computer-assisted image analyzer system consisting of a microscope (Olympus BH-2 Tokyo, Japan) equipped with a high-resolution video camera (JVC TK-890E, Japan). The numbers of neurons in hippocampal CA1, CA2, CA3, and gyrus dentatus regions were counted using a 6000 *μ* m 2 counting frame viewed through a 20 X Nikon lens at the monitor. The counting frame was placed randomly five times on the image analyzer system monitor, the numbers of neurons were counted (UTHSCA Image Tool for windows version 3.0 software), and the average was determined. Hippocampal neuron density then was calculated.

### 2.4. Hippocampal Leptin Receptor Expression

#### 2.4.1. RNA Isolation and cDNA Synthesis

Hippocampal tissue samples were each individually homogenized with Qiagen TissueLyser II homogenizer in 1 mL QIAzol lysis reagent, and total RNA was isolated using the RNeasy lipid tissue mini kit (Qiagen, GmbH, Germany) according to the manufacturer's instructions. DNA contamination was removed by optional on-column DNase-digestion using the RNase-Free DNase Set (Qiagen, GmbH, Germany). The quantity of total RNA was measured by the Nanodrop ND-1000 spectrophotometer (NanoDrop Technologies, Wilmington, DE, USA). The integrity of total RNA was analyzed in a MOPS-buffered 1.2% agarose gel containing formaldehyde and confirmed by visualization with EtBr staining and UV illumination. The absence of contaminating genomic DNA was also confirmed by setting reverse transcriptase minus (RT-) negative control reaction. The cDNA synthesis was performed with a RevertAid First Strand cDNA synthesis kit (Thermo Fisher Scientific Inc.) using 2 *μ*g of total RNA according to the manufacturer's instructions. 

### 2.5. Real-Time RT-PCR

Specific rat primer pairs and probes for reference and target genes for real-time polymerase chain reaction (PCR) analysis were designed using the ProbeFinder Software, which is available online (http://www.universalprobelibrary.com/). B-actin was used as the reference gene. The UPL (universal probe library) probe number for the leptin receptor gene was 118, and the primer sequences were as follows: Forward: 5′-TGTCAGAAATTCTATGTGGTTTTGT-3′; Reverse: 5′-TTGGATAGGCCAGGTTAAGTG (76 bp). The UPL probe number for *β*-actin gene was 115, and the primer sequences were as follows: Forward: 5′-CTAAGGCCAACCGTGAAAAG-3′; Reverse: 5′-GCCTGGATGGCTACGTACA-3′ (79 bp). All PCR reactions were performed using LightCycler 480.

Probes Master in a 20 *μ*L reaction mixture (2 *μ*L cDNA; 10 *μ*L LightCycler 480 Probes Master, and a 2 *μ*L (10 mM) each specific gene primer pairs and upl probes) in a LightCycler 480 Instrument, 96-well format (Roche Diagnostics, Indianapolis, Ind.). The PCR program was initiated at 95°C for 10 minutes before 40 cycles, each for 10 seconds at 95°C, 30 seconds at 60°C, 1 second at 70°C, and 30 seconds at 40°C for cooling, were conducted. Negative control, consisting of real-time RT-PCR reaction mixture using sterile distilled water instead of template RNA, was included in each batch of PCR. In addition, repeatability and reproducibility of the assays were assessed by performing 3 replicas. Each amplification cycle was analyzed with the LightCycler 480 software.

## 3. Statistical Evaluation

Differences between days in learning tests were evaluated with GLM-repeated measures in SPSS program, and differences between groups were evaluated with Mann-Whitney *U* test, which is a nonparametric test. Differences between groups in terms of all measurement results were also evaluated with Mann-Whitney *U* test. 

## 4. Results

**Figure 1:**
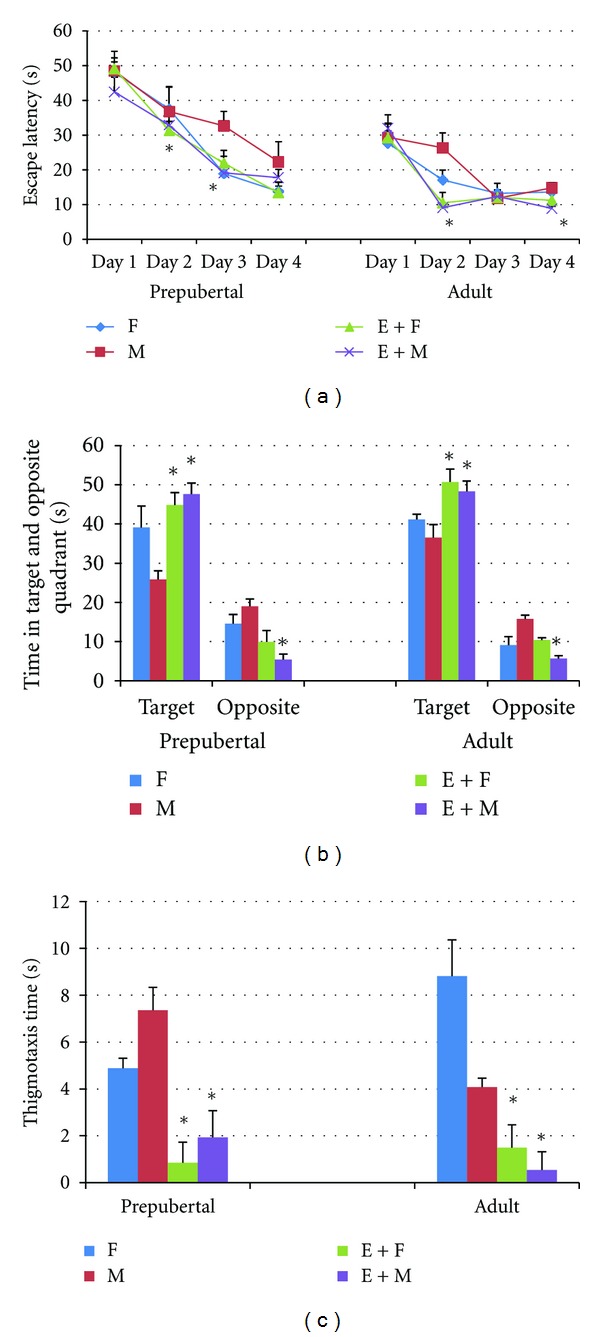
The Morris Water Maze performance of the groups. (a) The daily change in escape latency. (b) Time spent in the target and opposite quadrant in probe trial. (c) Thigmotaxis time in probe trial. *P < 0.05 compared to the other groups. F: control female pups, M: control male pups, E + F: female pups whose mothers exercised during pregnancy, E + M: male pups whose mothers exercised during pregnancy.

**Figure 2:**
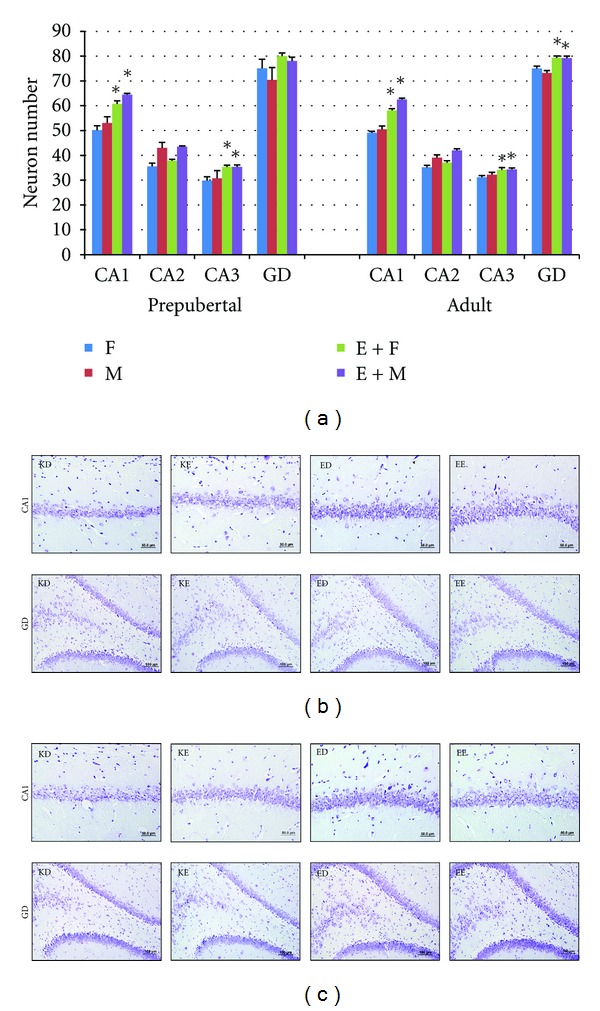
Hippocampal neuron density of the groups. (a) Quantitative evaluation of neuronal densities of CA1, CA2, and CA3 regions of hippocampus and gyrus dentatus. (b) Light microscope images of prepubertal pups (Cresyl violet staining). (c) Light microscope images of adult pups (Cresyl violet staining). *P < 0.05 compared to other groups. F: control female pups, M: control male pups, E + F: female pups whose mothers exercised during pregnancy, E + M: male pups whose mothers exercised during pregnancy. GD: gyrus dentatus.

**Figure 3:**
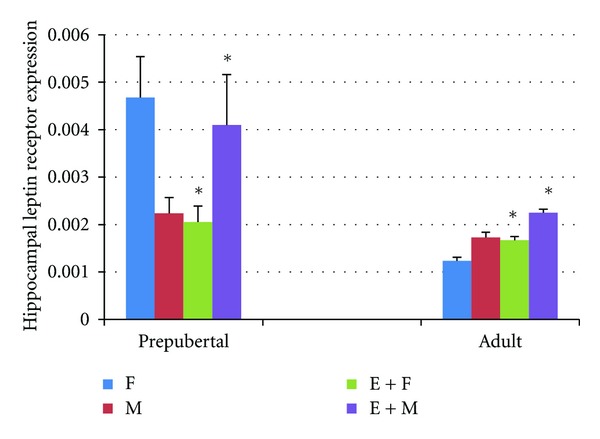
Hippocampal leptin receptor expression of the groups. *P < 0.05 compared to other groups. F: control female pups, M: control male pups, E + F: female pups whose mothers exercised during pregnancy, E + M: male pups whose mothers exercised during pregnancy.

It was observed in this study that the duration spent for finding the platform shortened and reached a steady level in all rats during the 4-day learning test process. All of the prepubertal and adult pups whose mothers performed regular exercise during pregnancy learned the place of the platform in MWM tests in a shorter time as compared to the control group; however, there were differences between groups in learning process. Among the pups whose mothers exercised during pregnancy, prepubertal females learned the place of the platform on the 2nd day (*P* < 0.05), prepubertal males learned the place on the 3rd day (*P* < 0.05), adult females learned the place on the 2nd day (*P* < 0.05), and adult males learned the place on the 2nd and the 4th days (*P* < 0.006) of the learning test, which were shorter in comparison with those measured in the control group (Figure 1(a)). It was seen that in probe trial, all pups whose mothers exercised during pregnancy spent more time in the target quadrant and less time in the opposite quadrant (*P* < 0.05) (Figure 1(b)). It was also determined that, in these groups, thigmotaxis periods were significantly shorter as compared to the control group (*P* < 0.05) (Figure 1(c)). In histological evaluation, the number of neurons increased CA1 (*P* < 0.005) and CA3 (*P* < 0.006) in prepubertal female pups of rats who exercised during pregnancy, CA1 (*P* < 0.004) and CA3 (*P* < 0.004) in their male offsprings, CA1 (*P* < 0.003), CA3 (*P* < 0.05), and GD (*P* < 0.05) in their adult female offsprings, and CA1 (*P* < 0.004), CA3 (*P* < 0.05), and GD (*P* < 0.004) in their adult male offsprings (Figures 2(a), 2(b), and 2(c)). Hippocampal leptin receptor expression was found to be significantly higher in prepubertal male (*P* < 0.05), adult female (*P* < 0.007), and adult male (*P* < 0.011) pups whose mothers exercised during pregnancy as compared to the control group. On the other hand, hippocampal leptin expression in prepubertal females was significantly lower compared to the control group (*P* < 0.002) (Figure 3).

## 5. Discussion

In this study, the CA1 and CA3 cell counts were significantly higher in prepubertal female and male pups of rats who exercised during pregnancy; CA1, CA3, and GD cell counts were higher in their adult female and male pups as compared to the control group. Furthermore, it was also determined that hippocampal leptin receptor expression increased in prepubertal male and all adult pups of mothers who exercised during pregnancy. Maternal exercise during pregnancy improved the learning process both in prepubertal and in adult offsprings as compared to the control group, they become more successful in the probe trial, they spent more time in the target quadrant and less time in the opposite quadrant, and they made less mistakes. Moreover, the thigmotaxis period which is a sign of anxiety was shorter in all pups of rats who exercised during pregnancy. The results of one of our previous studies also revealed that maternal exercise decreased the anxiety level of offsprings [32]. It is known that exercise has positive effects on cognitive functions. During exercise, oxygen saturation [34] and angiogenesis [35] increase in the brain. Hippocampus is an area of the brain playing a key role in learning and memory [5]. It is shown that acute voluntary or forced exercise increases neuronal activity in hippocampus [36]. It is also thought that neurotransmitters such as serotonin, norepinephrine, acetylcholine, and GABA (gamma aminobutyric acid) increase with exercise and regulate regions of hippocampus related to memory [37]. Besides, various studies concluded that exercise increases some neurotrophic factors such as BDNF, IGF-1 and vascular endothelial growth factor (VEGF) [16, 38, 39]. The effects of maternal exercise on the intrauterine hippocampus development are highly complex. Parnpiansil et al. [16] showed that maternal exercise during pregnancy increased the spatial learning of offsprings and hippocampal BDNF expression. It is recently known that leptin, from the cytokine family, also positively affects learning and memory by means of its receptors in hippocampus [40]. It is suggested that synaptic plasticity is regulated by leptin and leptin receptors in rats [41, 42]. It is shown that leptin receptors are expressed especially in the CA1 subfield, of the hippocampus [41, 43], facilitate presynaptic neurotransmitter secretion in hippocampal CA1 subfield, and increase the sensitivity of postsynaptic CA1 neurons against neurotransmitters [44]. By increasing NMDA receptors, leptin contributes the conversion of LTP into spatial memory in CA1 region of hippocampus [40, 45]. Spatial memory behaviors related to hippocampus [43, 46] and short-term potentiation (STP) [43] decreased in rodents which have leptin receptor mutation (db/db mice or fa/fa rats). Direct injection of leptin into hippocampus increases learning and memory performance [44, 47] and facilitates LTP [42]. Leptin is thought to facilitate the conversion of STP into LTP [41]. CA1 neurons of hippocampus are necessary for spatial learning and memory [48]. It is known that CA1 subfield in hippocampus contributes to LTP [45]. CA1 neurons take information from entorhinal cortex or CA3 subfield and affect the learning process [11]. CA3 is connected to CA1 subfield by means of Schaffer Collaterals cycle, while CA1 outputs extend to subiculum, entorhinal cortex, and prefrontal cortex [49]. It is observed that rats which have lesion in hippocampus CA3 subfield and have a healthy CA1 subfield were successful in the learning process in MWM test but they swam aimlessly in the probe trial when the information is reclaimed [48]. Consequently, a healthy CA3 and CA1–CA3 connection is an obligation for reference memory. An important result of this study is that maternal aerobic exercise caused an increase in neurons in CA1 and CA3 hippocampal areas of offsprings in their adult periods and an increase in hippocampal leptin receptor expression. It may be thought that maternal aerobic exercise during pregnancy positively affects the brain development of offsprings in their adult periods, as well. However, the fact that leptin receptor expression of prepubertal female pups was low and, on the other hand, hippocampal CA1 and CA3 neuron numbers were high and rats were more successful in learning memory tests in comparison with those in the control group gives the idea that maternal exercise during pregnancy has an effect on the generation or secretion of other neurotrophic factors for the brain development of offsprings. 

In this study, a significant increase was found in number of neurons in GD along with the neurogenesis in CA1 and CA3 of the adult pups whose mothers exercised during pregnancy. The studies conducted revealed that postnatal hippocampal neurogenesis is correlated with learning ability and memory capacity [13]. It is shown that new neurons are continuously produced in the GDs of adult mammals [50], especially enriched environment and physical exercise increase neurogenesis in GD [12, 38] and prevent neuron activity precursors decrease depending on the age [51]. In this study, increase in neurons in the GD of adult pups whose mothers exercised during pregnancy proves that maternal exercise during pregnancy also contributes to the hippocampal plasticity of offsprings in adult period.

In conclusion, it is seen that maternal aerobic exercise during pregnancy increases the hippocampal neurogenesis, hippocampal leptin receptor expression except prepubertal female pups, and learning memory performances in both prepubertal and adult pups. However, the fact that leptin expression was low in females in the prepubertal period and, on the other hand, learning memory performances and hippocampal neuron numbers were high gives the idea that other neurotrophic factors may also play a role in learning and memory of females in prepubertal period. How effects of leptin on synaptic plasticity are affected by other hormones and growth factors has been still not clear.Further studies are needed to elucidate the role of leptin and other neurotrophic factors' receptors on the mechanism of exercise to affect the functions of the hippocampus.

## References

[1] Cotman CW, Engesser-Cesar C (2002). Exercise enhances and protects brain function. *Exercise and Sport Sciences Reviews*.

[2] Swain RA, Harris AB, Wiener EC (2003). Prolonged exercise induces angiogenesis and increases cerebral blood volume in primary motor cortex of the rat. *Neuroscience*.

[3] Black JE, Isaacs KR, Anderson BJ, Alcantara AA, Greenough WT (1990). Learning causes synaptogenesis, whereas motor activity causes angiogenesis, in cerebellar cortex of adult rats. *Proceedings of the National Academy of Sciences of the United States of America*.

[4] Pysh JJ, Weiss GM (1979). Exercise during development induces an increase in Purkinje cell dendritic tree size. *Science*.

[5] Howe ML, Stones MJ, Brainerd CJ (1990). *Cognitive and Behavioral Performance Factors in a Typical Aging*.

[6] Uysal N, Tugyan K, Kayatekin BM (2005). The effects of regular aerobic exercise in adolescent period on hippocampal neuron density, apoptosis and spatial memory. *Neuroscience Letters*.

[7] Rezaie P, Trillo-Pazos G, Everall IP, Male DK (2002). Expression of *β*-chemokines and chemokine receptors in human fetal astrocyte and microglial co-cultures: potential role of chemokines in the developing CNS. *GLIA*.

[8] Marco EM, Adriani W, Llorente R, Laviola G, Viveros MP (2009). Detrimental psychophysiological effects of early maternal deprivation in adolescent and adult rodents: altered responses to cannabinoid exposure. *Neuroscience and Biobehavioral Reviews*.

[9] Tugyan K, Uysal N, Ozdemir D (2006). Protective effect of melatonin against maternal deprivation-induced acute hippocampal damage in infant rats. *Neuroscience Letters*.

[10] Rosenzweig MR, Bennett EL (1996). Psychobiology of plasticity: effects of training and experience on brain and behavior. *Behavioural Brain Research*.

[11] O'Keefe J, Nadel L (1978). *The Hippocampus as a Cognitive Map*.

[12] Van Praag H, Christie BR, Sejnowski TJ, Gage FH (1999). Running enhances neurogenesis, learning, and long-term potentiation in mice. *Proceedings of the National Academy of Sciences of the United States of America*.

[13] Gould E, Tanapat P (1999). Stress and hippocampal neurogenesis. *Biological Psychiatry*.

[14] Uysal N, Ozdemir D, Dayi A, Yalaz G, Baltaci AK, Bediz CS (2005). Effects of maternal deprivation on melatonin production and cognition in adolescent male and female rats. *Neuroendocrinology Letters*.

[15] Bungum TJ, Peaslee DL, Jackson AW, Perez MA (2000). Exercise during pregnancy and type of delivery in nulliparae. *Journal of Obstetric, Gynecologic, and Neonatal Nursing*.

[16] Parnpiansil P, Jutapakdeegul N, Chentanez T, Kotchabhakdi N (2003). Exercise during pregnancy increases hippocampal brain-derived neurotrophic factor mRNA expression and spatial learning in neonatal rat pup. *Neuroscience Letters*.

[17] Ahima RS (2000). Leptin and the neuroendocrinology of fasting. *Frontiers of Hormone Research*.

[18] Morash B, Li A, Murphy PR, Wilkinson M, Ur E (1999). Leptin gene expression in the brain and pituitary gland. *Endocrinology*.

[19] Schwartz MW, Peskind E, Raskind M, Boyko EJ, Porte D (1996). Cerebrospinal fluid leptin levels: relationship to plasma levels and to adiposity in humans. *Nature Medicine*.

[20] Banks WA, Kastin AJ, Huang W, Jaspan JB, Maness LM (1996). Leptin enters the brain by a saturable system independent of insulin. *Peptides*.

[21] Couce ME, Burguera B, Parisi JE, Jensen MD, Lloyd RV (1997). Localization of leptin receptor in the human brain. *Neuroendocrinology*.

[22] Elmquist JK, Bjorbaek C, Ahima RS, Flier JS, Saper CB (1998). Distributions of leptin receptor mRNA isoforms in the rat brain. *Journal of Comparative Neurology*.

[23] Håkansson M-L, Brown H, Ghilardi N, Skoda RC, Meister B (1998). Leptin receptor immunoreactivity in chemically defined target neurons of the hypothalamus. *Journal of Neuroscience*.

[24] Bereiter DA, Jeanrenaud B (1979). Altered neuroanatomical organization in the central nervous system of the genetically obese (ob/ob) mouse. *Brain Research*.

[25] Sena A, Sarliève LL, Rebel G (1985). Brain myelin of genetically obese mice. *Journal of the Neurological Sciences*.

[26] Steppan CM, Swick AG (1999). A role for leptin in brain development. *Biochemical and Biophysical Research Communications*.

[27] Ahima RS, Bjorbæk C, Osei S, Flier JS (1999). Regulation of neuronal and glial proteins by leptin: implications for brain development. *Endocrinology*.

[28] Anderson MF, Åberg MAI, Nilsson M, Eriksson PS (2002). Insulin-like growth factor-I and neurogenesis in the adult mammalian brain. *Developmental Brain Research*.

[29] Holmes MM, Galea LAM, Mistlberger RE, Kempermann G (2004). Adult hippocampal neurogenesis and voluntary running activity: circadian and dose-dependent effects. *Journal of Neuroscience Research*.

[30] Hummel KP, Dickie MM, Coleman DL (1966). Diabetes, a new mutation in the mouse. *Science*.

[31] Stranahan AM, Lee K, Martin B (2009). Voluntary exercise and caloric restriction enhance hippocampal dendritic spine density and BDNF levels in diabetic mice. *Hippocampus*.

[32] Aksu I, Baykara B, Ozbal S (2012). Maternal treadmill exercise during pregnancy decreases anxiety and increases prefrontal cortex VEGF and BDNF levels of rat pups in early and late periods of life. *Neuroscience Letters*.

[33] Paxinos G, Watson C (1982). *The Rat Brain in Stereotaxic Coordinates*.

[34] Kramer AF, Hahn S, Cohen NJ (1999). Ageing, fitness and neurocognitive function. *Nature*.

[35] Kleim JA, Cooper NR, VandenBerg PM (2002). Exercise induces angiogenesis but does not alter movement representations within rat motor cortex. *Brain Research*.

[36] Bland BH, Bird J, Jackson J, Natsume K (2006). Medial septal modulation of the ascending brainstem hippocampal synchronizing pathways in the freely moving rat. *Hippocampus*.

[37] Ma Q (2008). Beneficial effects of moderate voluntary physical exercise and its biological mechanisms on brain health. *Neuroscience Bulletin*.

[38] Trejo JL, Carro E, Torres-Alemán I (2001). Circulating insulin-like growth factor I mediates exercise-induced increases in the number of new neurons in the adult hippocampus. *Journal of Neuroscience*.

[39] Fabel K, Fabel K, Tam B (2003). VEGF is necessary for exercise-induced adult hippocampal neurogenesis. *European Journal of Neuroscience*.

[40] Shioda S, Funahashi H, Nakajo S, Yada T, Maruta O, Nakai Y (1998). Immunohistochemical localization of leptin receptor in the rat brain. *Neuroscience Letters*.

[41] Shanley LJ, Irving AJ, Harvey J (2001). Leptin enhances NMDA receptor function and modulates hippocampal synaptic plasticity. *Journal of Neuroscience*.

[42] Wayner MJ, Armstrong DL, Phelix CF, Oomura Y (2004). Orexin-A (Hypocretin-1) and leptin enhance LTP in the dentate gyrus of rats in vivo. *Peptides*.

[43] Li XL, Aou S, Oomura Y, Hori N, Fukunaga K, Hori T (2002). Impairment of long-term potentiation and spatial memory in leptin receptor-deficient rodents. *Neuroscience*.

[44] Oomura Y, Hori N, Shiraishi T (2006). Leptin facilitates learning and memory performance and enhances hippocampal CA1 long-term potentiation and CaMK II phosphorylation in rats. *Peptides*.

[45] Bliss TVP, Collingridge GL (1993). A synaptic model of memory: long-term potentiation in the hippocampus. *Nature*.

[46] Winocur G, Greenwood CE, Piroli GG (2005). Memory impairment in Obese Zucker rats: an investigation of cognitive function in an animal model of insulin resistance and obesity. *Behavioral Neuroscience*.

[47] Farr SA, Banks WA, Morley JE (2006). Effects of leptin on memory processing. *Peptides*.

[48] Brun VH, Otnæss MK, Molden S (2002). Place cells and place recognition maintained by direct entorhinal-hippocampal circuitry. *Science*.

[49] Amaral DG, Witter MP (1995). *The Hippocampal Formation: The Rat Nervous System*.

[50] Eriksson PS, Perfilieva E, Björk-Eriksson T (1998). Neurogenesis in the adult human hippocampus. *Nature Medicine*.

[51] Kronenberg G, Lippoldt A, Kempermann G (2006). Two genetic rat models of arterial hypertension show different mechanisms by which adult hippocampal neurogenesis is increased. *Developmental Neuroscience*.

